# Is natural (6*S*)-5-methyltetrahydrofolic acid as effective as synthetic folic acid in increasing serum and red blood cell folate concentrations during pregnancy? A proof-of-concept pilot study

**DOI:** 10.1186/s13063-020-04320-3

**Published:** 2020-05-05

**Authors:** Kelsey M. Cochrane, Chantal Mayer, Angela M. Devlin, Rajavel Elango, Jennifer A. Hutcheon, Crystal D. Karakochuk

**Affiliations:** 1 Food, Nutrition and Health, Faculty of Land and Food Systems, The University of British Columbia, 2205 East Mall, Vancouver, BC V6T 1Z4 Canada; 2 BC Children’s Hospital Research Institute, 950 West 28th Avenue, Vancouver, BC V5Z 4H4 Canada; 3 Department of Obstetrics and Gynaecology, University of British Columbia, 4500 Oak Street, Vancouver, BC V6H 3N1 Canada; 4 Department of Paediatrics, Faculty of Medicine, The University of British Columbia, 4480 Oak Street, Vancouver, BC V6H 3V4 Canada; 5 School of Population and Public Health, Faculty of Medicine, The University of British Columbia, 2206 East Mall, Vancouver, BC V6T 1Z3 Canada

**Keywords:** Nutrition, Folate, Folic acid, Pregnancy, Neural tube defects

## Abstract

**Background:**

North American health authorities recommend 0.4 mg/day folic acid before conception and throughout pregnancy to reduce the risk of neural tube defects. Folic acid is a synthetic form of folate that must be reduced by dihydrofolate reductase and then further metabolized. Recent evidence suggests that the maximal capacity for this process is limited and unmetabolized folic acid has been detected in the circulation. The biological effects of unmetabolized folic acid are unknown. A natural form of folate, (6*S*)-5-methyltetrahydrofolic acid (Metafolin®), may be a superior alternative because it does not need to be reduced in the small intestine. Metafolin® is currently used in some prenatal multivitamins; however, it has yet to be evaluated during pregnancy.

**Methods/design:**

This double-blind, randomized trial will recruit 60 pregnant women aged 19–42 years. The women will receive either 0.6 mg/day folic acid or an equimolar dose (0.625 mg/day) of (6*S*)-5-methyltetrahydrofolic acid for 16 weeks. The trial will be initiated at 8–21 weeks’ gestation (after neural tube closure) to reduce the risk of harm should (6*S*)-5-methyltetrahydrofolic acid prove less effective. All women will also receive a prenatal multivitamin (not containing folate) to ensure adequacy of other nutrients. Baseline and endline blood samples will be collected to assess primary outcome measures, including serum folate, red blood cell folate and unmetabolized folic acid. The extent to which the change in primary outcomes from baseline to endline differs between treatment groups, controlling for baseline level, will be estimated using linear regression. Participants will have the option to continue supplementing until 1 week postpartum to provide a breastmilk and blood sample. Exploratory analyses will be completed to evaluate breastmilk and postpartum blood folate concentrations.

**Discussion:**

This proof-of-concept trial is needed to obtain estimates of the effect of (6*S*)-5-methyltetrahydrofolic acid compared to folic acid on circulating biomarkers of folate status during pregnancy. These estimates will inform the design of a definitive trial which will be powered to assess whether (6*S*)-5-methyltetrahydrofolic acid is as effective as folic acid in raising blood folate concentrations during pregnancy. Ultimately, these findings will inform folate supplementation policies for pregnant women.

**Trial registration:**

ClinicalTrials.gov, ID: NCT04022135. Registered on 14 July 2019.

## Background

### Overview of folate

Folate refers to a group of water-soluble B-vitamins which play a critical role in deoxyribonucleic acid (DNA) synthesis and methylation reactions [1]. All folate vitamers consist of a common parent structure, but differ based on whether the pteridine ring is in an oxidized or a reduced state, the addition of one-carbon substituents at positions N5 and/or N10 and number of glutamic acid residues [1, 2]. Food folates are reduced and have a polyglutamate tail consisting of up to nine glutamate residues [1, 3]. Folic acid is a synthetic form of folate which is oxidized and contains one glutamic acid residue; this is the most stable form. Therefore, folic acid is used in food fortification and most supplements [1–3].

**Fig. 1:**
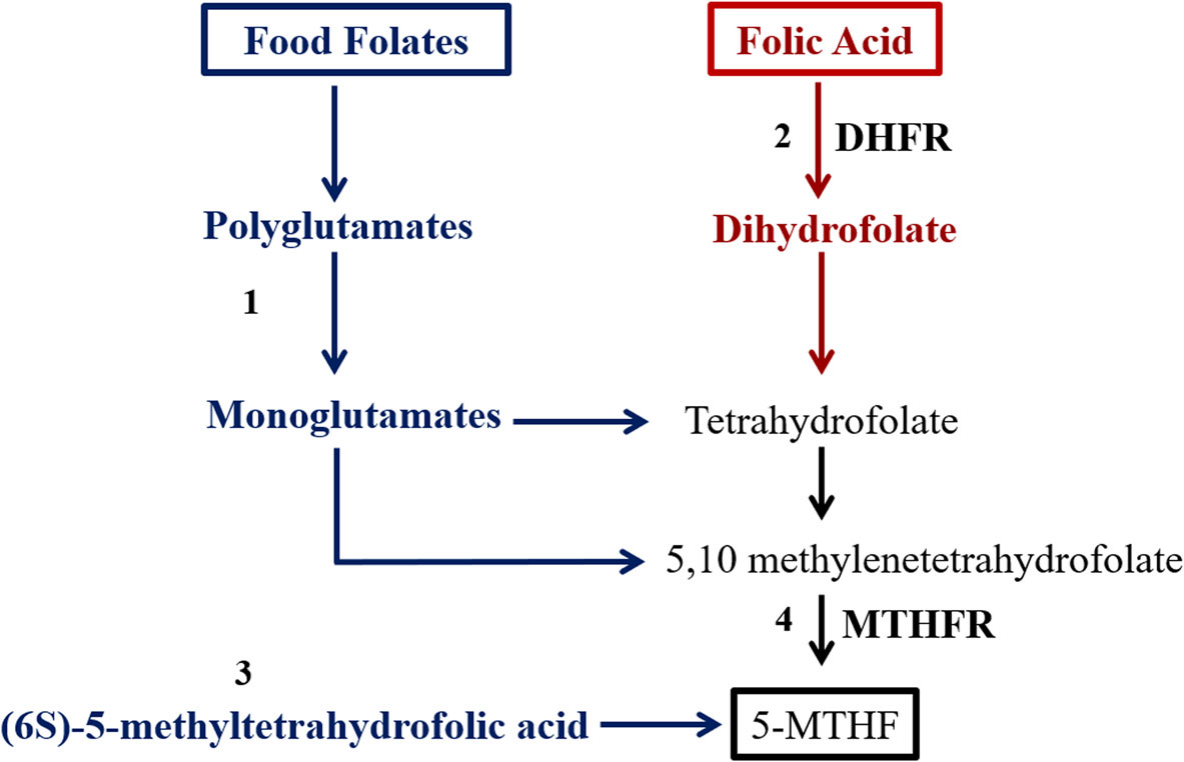
Intestinal absorption of natural folate and folic acid. Legend: (1) food folates are hydrolyzed during digestion to the monoglutamate form; (2) Folic acid must be reduced to tetrahydrofolate in a two-step reaction by DHFR; (3) supplements in the natural form of folate, (6S)-5-methyltetrahydrofolic acid, may enter circulation directly for use in the body as 5-MTHF; and (4) both food folates and folic acid are ultimately metabolized to 5-MTHF by MTHFR

Bioavailability and metabolism of folates differ due to their respective chemical structures (Fig. 1) [1, 3]. Food folates are estimated to be about 50% bioavailable, as they must be hydrolyzed during digestion in the gut by a brush-border hydrolase [1, 4]. Conversely, folic acid is a monoglutamate and can be absorbed as such [2]. Under fasting conditions, folic acid is almost 100% bioavailable and when consumed with food, it is approximately 85% bioavailable [1, 4]. Ultimately, all forms of folate are metabolized to 5-methyltetrahydrofolate (5-MTHF) [2]. However, unlike food folates, folic acid must first be reduced to tetrahydrofolate in a two-step reaction via dihydrofolate reductase (DHFR). Tetrahydrofolate is then methylated to 5-MTHF via methylenetetrahydrofolate reductase (MTHFR) [2]. A natural form of folate (Metafolin®) is currently used in some nutrition supplements in place of folic acid. Metafolin® is a naturally occurring calcium salt of (6*S*)-5-methyltetrahydrofolic acid [2, 5]. It does not require reduction by DHFR and can enter the circulation directly for use in the body [2].

### Periconceptional folate requirements

Neural tube defects (NTDs) are severe, irreversible congenital anomalies which occur in the third to fourth week after conception [6]. Although their etiology remains unclear, numerous clinical trials have demonstrated that folic acid supplementation is effective in reducing the risk of NTDs [7–10]. North American guidelines recommend that all women at low risk for a NTD-affected pregnancy (which encompasses approximately 95% of pregnancies) supplement with 0.4 mg/day folic acid, starting 2–3 months before conception and throughout pregnancy [7, 10]. Due to many pregnancies being unplanned, several countries, including Canada and the United States, have introduced mandatory folic acid fortification of white flour, enriched pasta and cornmeal at a rate which aims to provide 0.1 mg/day [11, 12]. Folic acid food fortification has been greatly successful in reducing the prevalence of NTDs; rates of NTD-subtype spina bifida decreased by approximately one half (53% reduction observed in Canada and 41% in the United States) and others (anencephaly and encephalocele) by approximately one third [12, 13].

Most pregnant women in countries that use fortification consume greater than 0.4 mg/day folic acid due to the combination of food fortification and prenatal multivitamins (which typically contain 0.6–1.0 mg folic acid per dose) [14]. In a Canadian pregnancy cohort (*n* = 1533), 87% of women had total folic acid intakes (supplements and fortified foods) greater than the upper limit (1.0 mg/day) [15]. Studies in Canada and the United States have shown that population-wide blood folate levels have increased substantially following fortification [16, 17]. In two Toronto-based pregnancy cohorts, the mean red blood cell (RBC) folate was > 2300 nmol/L at 12–16 weeks’ gestation and at 36 weeks’ gestation, regardless of supplementation regimen [18, 19]. RBC folate > 1090 nmol/L is indicative of intakes > 2.1 mg dietary folate equivalents (DFEs) per day [17]. National surveys have identified that since the initiation of food fortification, the prevalence of folate deficiency (RBC folate < 305 nmol/L) has decreased from 38% to 5% in the United States and is essentially non-existent in Canada (< 1%) [16, 17, 20].

High intakes of folic acid are concerning because it appears that DHFR activity is limited [21–23], and the metabolic effects of unmetabolized folic acid (UMFA) are unknown. There is an acute appearance of plasma UMFA following oral intakes of folic acid > 0.2 mg, suggesting that intestinal absorptive capacity becomes saturated at this dose [24–27]. Following single doses of folic acid, it appears that the body can clear plasma UMFA via mechanisms including tissue uptake and urinary excretion [28, 29]. However, in two studies which provided folic acid (0.4 mg/day) for 8 and 14 weeks, UMFA was detected in fasting plasma samples [27, 30], suggesting that with regular daily intake, clearance mechanisms become saturated as well [28, 31]. This phenomenon was again observed in a Toronto-based study which evaluated the effects of 30-week folic acid supplementation (1.1 mg vs 5 mg) among non-pregnant women of reproductive age [28]. The proportion of women with detectable levels of UMFA increased following supplementation in both groups; however, concentrations were variable between participants and increases were not sustained [28]. This supports findings that DHFR capacity is variable in humans, but suggests that there are mechanisms which limit systemic exposure to circulating UMFA following high-dose, long-term folic acid supplementation [28]. Nonetheless, it appears that folic acid intake in Canadian pregnant women likely exceeds the physiologic capacity to reduce and utilize it or to clear it from plasma [32]. A Canadian prospective-cohort study found plasma UMFA detectable in > 90% of maternal and newborn (cord blood) samples (*n* = 368) [19]. Additionally, studies which have assessed breastmilk in Canadian women detected UMFA in 96% of milk samples [33, 34].

The presence of UMFA in breastmilk is concerning because it may impair the bioavailability of folate in milk [35, 36]. Folate-binding protein (FBP) in breastmilk has a higher affinity for folic acid than reduced folate forms; after ingestion, folic acid may be less readily released by FBP as it passes through the gastrointestinal tract, reducing subsequent absorption and potentially impacting the infant’s folate status [33]. Although total breastmilk folate concentrations are unaffected by maternal folate status (except in cases of severe deficiency) [34, 37], the proportion of breastmilk folate as folic acid may be influenced [33]. In a recent retrospective cohort study (*n* = 561), women who reported supplementing with > 0.4 mg/day folic acid had proportionally higher UMFA in breastmilk than women who reported supplementing with < 0.4 mg/day (50% as compared to 25%, respectively) [33]. The correlation, if any, between maternal plasma UMFA and breastmilk UMFA is unknown [34].

Natural (6*S*)-5-methyltetrahydrofolic acid is approved by Health Canada as a source material of folate [5] but it has never been evaluated in pregnancy. Previous studies have demonstrated that (6*S*)-5-methyltetrahydrofolic acid is more effective than folic acid in increasing serum and RBC folate in non-pregnant women of childbearing age [38–41] and lactating women [18]. (6*S*)-5-methyltetrahydrofolic acid may be a superior alternative to folic acid because it does not require reduction via DHFR, but it is critical to ensure that it can maintain serum and RBC folate concentrations throughout pregnancy in order to adequately supply the growing fetus. Additionally, when 0.4 mg folic acid was compared to (6*S*)-5-methyltetrahydrofolic acid during lactation, no difference in total breastmilk folate or proportion of UMFA in milk was found [34]. However, given recent findings that 0.4 mg/day folic acid may be a threshold for increasing the proportion of folic acid in milk from 25 to 50% [33], further investigation is warranted. Thus, we aim to address these gaps in the current literature by conducting a pilot study which will inform the design of a definitive trial investigating whether (6*S*)-5-methyltetrahydrofolic acid is as effective as synthetic folic acid in increasing serum and RBC folate concentrations during pregnancy and following delivery, while resulting in lower maternal plasma and breastmilk UMFA.

## Methods/design

### Study design

**Fig. 2:**
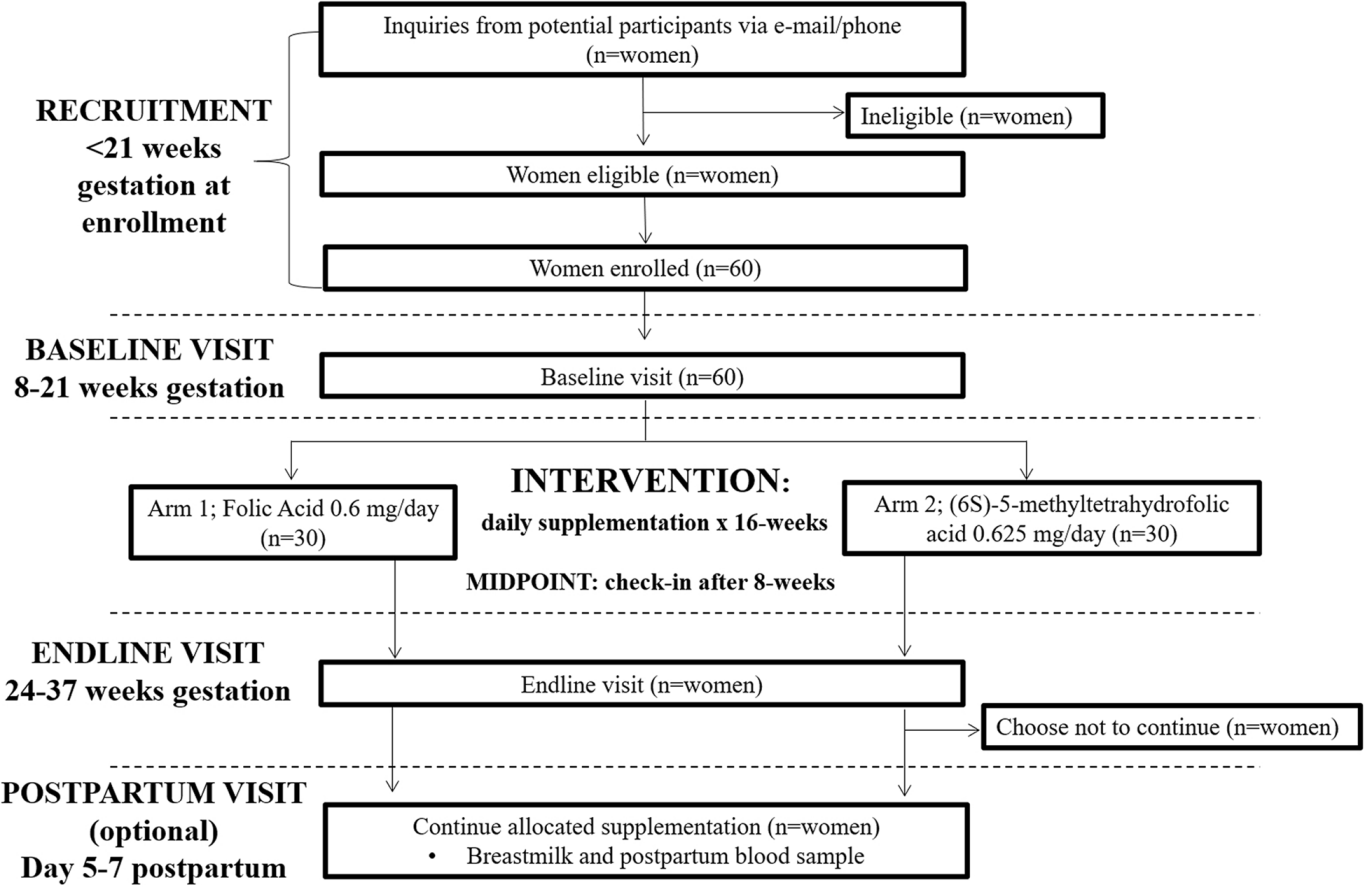
Participant flow diagram

**Fig. 3:**
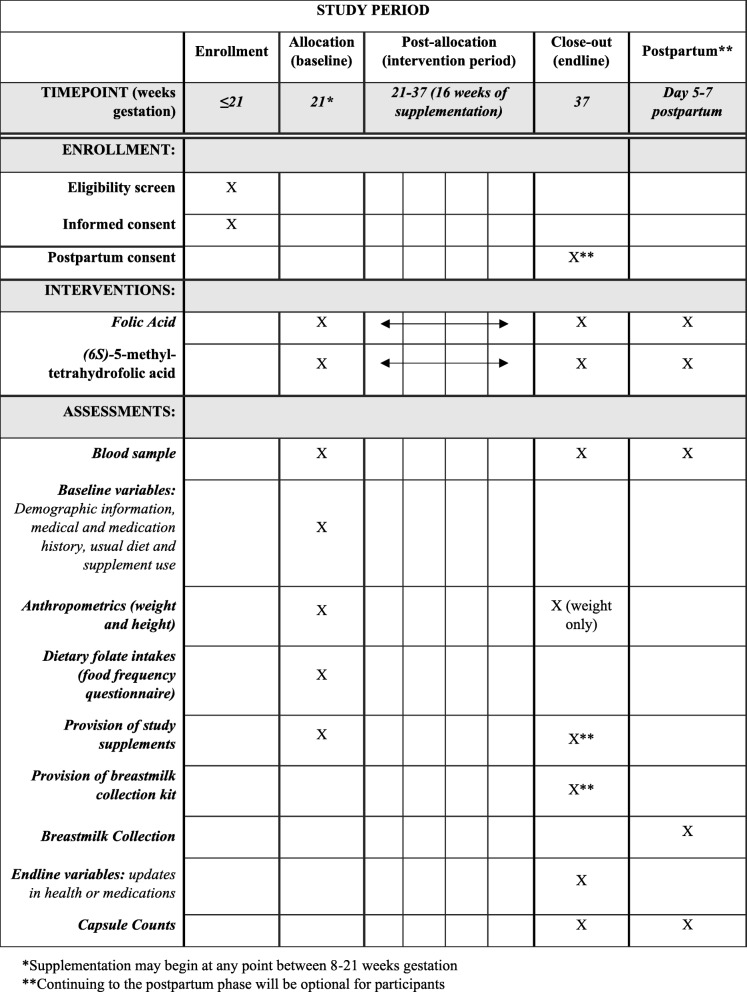
Standard Protocol Items: Recommendation for Interventional Trials (SPIRIT) schedule of enrollment, interventions and assessments

In this two-arm, double-blind, randomized trial, we will recruit 60 pregnant women aged 19–42 years living in Vancouver, BC, Canada. Participants will be randomized to supplement daily for 16 weeks with either 0.6 mg/day folic acid or an equimolar dose (0.625 mg/day) of (6*S*)-5-methyltetrahydrofolic acid (Fig. 2). The 0.6-mg dose will match levels found in leading commercial prenatal vitamin brands (Materna®), thus increasing the generalizability of our findings. The 16-week intervention was based on the estimated half-life of RBC folate and intends to enable the measurement of long-term folate status in RBCs (past 12–16 weeks) [42]. All participants will also receive a prenatal multivitamin not containing any folate, to ensure the adequacy of other nutrients (e.g., iron) during pregnancy. Fasting venous blood samples will be collected at baseline and endline to measure primary outcome measures, including serum folate, RBC folate and plasma UMFA; exploratory measures will include plasma *S-*adenosyl-methionine, *S-*adenosyl-homocysteine, total homocysteine, total cysteine, methionine, vitamin B_12_, pyridoxal-5-phosphate, free choline and betaine. Genotyping of the *MTHFR* (677 C > T, *rs*1801133, and 1298 A > C, *rs*1801131) and *DHFR* (*rs*1643649 and *rs*70991108) variants and a complete blood count will also be determined. Women will have the option to continue into the postpartum (exploratory) phase of the study, where at their endline visit they will be given more supplements to take until approximately 1 week postpartum. At days 5–7 postpartum, the women will provide a small (3-mL) breastmilk sample and a non-fasting venous blood sample. Outcomes evaluated in the breastmilk will include folate forms (UMFA, THF, 5-methyl-THF, 5-formyl-THF and 5,10-methenyl-THF) and milk FBP. Outcomes evaluated in the postpartum blood sample will include RBC folate and plasma UMFA. The study protocol has been developed in accordance with the 2013 Standard Protocol Items: Recommendation for Interventional Trials (SPIRIT) guidelines (Fig. 3) [43]. See Additional file 1 (SPIRIT Checklist) for further details.

The primary objective of this pilot study is to gain estimates for the change in serum folate, RBC folate and UMFA following supplementation with (6*S*)-5-methyltetrahydrofolic acid and folic acid for 16 weeks of pregnancy to inform the design of an adequately powered definitive trial. The secondary objective is to obtain recruitment and participation rates to assist in the design and feasibility of a definitive trial. The exploratory objectives of the postpartum phase are to quantify the proportion of total breastmilk folate as folic acid in each group, evaluate the correlation between maternal postpartum plasma UMFA and breastmilk folic acid, and to evaluate RBC folate concentrations following delivery in each group.

### Participants, recruitment and informed consent

*Inclusion criteria*: (1) pregnant woman (singleton pregnancy); (2) living in the greater Vancouver area; (3) ≤ 21 weeks’ gestation at time of consent; (4) 19–42 years of age; and (5) able to provide informed consent.

*Exclusion criteria*: (1) pre-existing medical conditions known to impact maternal folate status (malabsorptive and inflammatory bowel diseases, active celiac disease, gastric bypass surgery, atrophic gastritis, epilepsy, advanced liver disease, kidney dialysis, type 1 or 2 diabetes mellitus, sickle cell trait/anemia) [7]; (2) lifestyle factors known to impact maternal folate status (current smoking, alcohol consumption, recreational drug use) [7]; (3) being medium to high risk for development of an NTD-affected pregnancy (applies to women or their male partner: personal or family history (parents or siblings) of other folate-sensitive congenital anomalies, personal NTD history or a previous NTD-affected pregnancy) [7]; (4) medications known to interfere with B-vitamin metabolism (chloramphenicol, methotrexate, metformin, sulfasalazine, phenobarbital, phenytoin, primidone, triamterene, barbiturates) [7]; (5) pre-pregnancy body mass index (BMI) ≥ 30 kg/m^2^; or (6) allergy to any study supplement ingredients.

#### Sample size

The sample size was calculated based on the primary objective to estimate the distribution (mean ± standard deviation) of change in primary outcomes (serum folate, RBC folate and UMFA) following 16 weeks of supplementation. To accomplish this, we will require 50 women (25 in each group); a sample of at least *n* = 50 is generally recognized as adequate for clinical research pilot studies [44]. A 20% inflation will be added to account for dropout participants or loss to follow-up; thus, a total of 60 women (30 in each group) will be recruited.

#### Recruitment

Posters will be displayed at the BC Women’s Hospital, in medical and prenatal clinics and in retail establishments for pregnant women (e.g., prenatal classes, fitness studios, maternity clothing stores) throughout Vancouver, BC, Canada. Study details will be shared with staff and health care professionals, including physicians, midwives, nurses and others as applicable. Advertising on social media (Facebook and Instagram) will be used and targeted towards pregnant women aged 19–42 years in the greater Vancouver, BC area.

#### Obtaining informed consent and confirming eligibility

Women interested in participating will contact the research coordinator (KMC) directly. At this time, study details, eligibility and informed consent will be described. Eligible participants who would like to be enrolled will be given a study ID and their baseline visit will be scheduled. Participants will be instructed to continue any current prenatal multivitamin/folate supplementation until their baseline visit. The optional postpartum phase of the study will be described in the informed consent form; however, participants who choose to continue past the primary 16 weeks will be given a separate consent form to sign.

### Study visits

Study visits will be facilitated in Vancouver, BC, Canada at the University of British Columbia, Food, Nutrition and Health Building and in the Clinical Research Evaluation Unit at the BC Children and Women’s Hospital. *Baseline* (8–21 weeks’ gestation): Both the participant and the research coordinator (KMC) will sign the informed consent form; a copy will be scanned and e-mailed to the participant following the study visit. Participants will complete a baseline questionnaire to capture medical and nutrition history and demographic data. A validated food frequency questionnaire will be used to calculate DFEs [45]. Weight and height will be taken using an electronic scale and stadiometer and recorded to 0.1 kg and 0.10 m, respectively. A 12-mL, 3-h fasting venous blood sample will be collected. Participants will receive their study supplements (either folic acid or (6*S*)-5-methyltetrahydrofolic acid) and be provided with a supplement diary to record daily intake. *Midpoint*: after 8 weeks, all women will be contacted to check-in on compliance, address any questions or concerns, and to schedule an endline visit. *Endline* (24–37 weeks’ gestation): a 12-mL, 3-h fasting venous blood sample will be collected, weight will be measured as described above and participants will complete an endline questionnaire to determine any changes in health status or medication use since baseline. Participants will return their supplement diary and all remaining capsules will be counted. *Postpartum*: at the endline visit, participants who are planning to breastfeed and who have a working household freezer may continue into the postpartum phase of the study. Once consent has been obtained using a second informed consent form, they will be given supplements to continue taking until approximately 1 week postpartum and a new supplement diary. Participants will be trained on how to collect a small (3-mL) breastmilk sample in their home at days 5–7 postpartum and given all supplies needed (described in more detail in the “Breastmilk collection and processing” section below). Participants will store their breastmilk sample in the freezer and notify the research coordinator when it is ready for pick up. Pick up will occur within 4 days of notification and a 12-mL venous blood sample will be taken at this time. The women will be instructed to take their folate supplement 2 h prior to collection of both breastmilk and blood samples in order to standardize the peak in plasma UMFA (and potentially uptake of UMFA into breastmilk; however, the timing of this process not well described).

### Study supplements

The folic acid and (6*S*)-5-methyltetrahydrofolic acid will be provided separately from the prenatal multivitamins. This decision was made because some women report intolerance when consuming prenatal multivitamins; this is typically due to the high iron content and resolves after the first trimester [46]. Should temporary intolerance arise, participants may skip or take a half dose of their multivitamin, while continuing to supplement with the full dose of folate. The bulk ingredients for the folic acid and prenatal multivitamins have been provided by Natural Factors (Coquitlam, BC, Canada). Bulk (6*S*)-5-methyltetrahydrofolic acid (Metafolin®) has been provided by Merck & Cie (Schaffhausen, Switzerland). All bulk ingredients have been compounded into vegetable-gel capsules at Natural Factors (Coquitlam, BC, Canada). Folic acid and (6*S*)-5-methyltetrahydrofolic acid capsules are identical in appearance to accommodate double-blinding. The prenatal multivitamin contains the same micronutrient formulation as WN Pharmaceuticals Ltd.® *Prenatal* (NPN 80025456), except the folic acid has been removed. A quality overall summary was completed for all study supplements by Natural Factors (Coquitlam, BC, Canada) and verified by Health Canada, with confirmation of folic acid and (6*S*)-5-methyltetrahydrofolic acid doses via high-performance liquid chromatography. A notice of authorization for the clinical trials application (Submission No. 244456) was provided by the natural and non-prescription health products directorate of Health Canada on 26 July 2019.

### Double-blinding, randomization and provision of supplement packs

An independent research assistant from Natural Factors (Coquitlam, BC, Canada) will assign blinding codes (A or B) to the folic acid and (6*S*)-5-methyltetrahydrofolic acid supplements. Folate supplement bottles will be labeled with “A” or “B” and indicate that “capsules are either folic acid or folate.” Both the participants and the primary research team will be blinded to the supplement allocations, which will be de-coded only once the final statistical analyses are completed. The randomization sequence will be computer-generated by an independent statistician, using blocks of four which each contain two participants per supplement group. The primary research team will be blinded to the randomization sequence. At each participant’s baseline visit, the research coordinator (KMC) will unblind the allocation (A or B) for that study ID only in order to provide the allocated study supplements. At baseline, participants will receive a supplement pack containing all capsules for the 16-week intervention; if participants choose to continue to the postpartum phase of the study, they will be given more supplements at their endline visit to continue taking until ~ 1 week postpartum.

### Concomitant medications

Once enrolled, there will be no restrictions on medication intake throughout the intervention period; however, all medications must be reported. The only exception will be folate/folic-acid-containing nutritional supplements, which will not be permitted throughout the intervention period.

### Strategies to enhance adherence

Pregnant women tend to be highly motivated and are generally interested in participating in clinical research trials [47]. As per a survey from the Public Health Agency of Canada, 94% of women in British Columbia supplement with prenatal multivitamins during pregnancy [48]. Therefore, participants will likely be accustomed to daily supplementation upon enrollment. The research coordinator (KMC) will contact each participant at midpoint (8 weeks after their baseline visit) to address any questions or concerns. Additionally, the supplement pack will allow for organized storage of supplements at home and the supplement diary will serve as a daily reminder to enhance adherence throughout the 16 weeks. For these reasons, we anticipate high adherence (> 90%) to the study protocol.

### Blood sample collection and processing

Venous blood samples (12 mL total) will be collected in a 6-mL ethylenediaminetetraacetic acid

(EDTA) tube, a 2-mL EDTA tube and a 4-mL serum tube (BD Biosciences, Franklin Lakes, NJ, USA) at baseline, endline and postpartum (if applicable) visits. Baseline and endline blood draws will occur following a 3-h fast; this timeframe is sufficient to reduce the confounding influence of recent folate intake on serum concentrations. The postpartum blood sample (~ 1 week postpartum) will be non-fasting, as this will not influence RBC folate and will reduce burden on participants. After collection, tubes will be shielded from light, inverted gently as per manufacturers recommendations, placed in a cooler and transported to the laboratory for immediate processing (within 2 h of collection).

For preparation of whole-blood hemolysate, whole blood (0.3 mL) will be removed from the 6-mL EDTA tube and diluted 1/11 by adding 3.0 mL of a 1% ascorbic acid solution and subsequently incubated at 37 °C for 30 min. The 6-mL EDTA tube will then be centrifuged at 3000 rpm for 15 min at 4 °C. Plasma will be collected and (for baseline and endline samples only) remaining contents of the 6-mL EDTA tube will be processed for isolation of peripheral blood mononuclear layer cells (PBMCs) with the SepMate PBMC isolation protocol (STEMCELL Technologies, Vancouver, BC, Canada). The 2-mL EDTA tube will be used for a complete blood count. The 4-mL serum tube will be left at room temperature for ~ 30 min (until clotted) and then centrifuged at 3000 rpm for 15 min at 4 °C; serum will be collected. All aliquots will be stored at − 80 °C until further analyzed.

### Breastmilk collection and processing

The women will be instructed to collect breastmilk on days 5–7 postpartum between 13:00 and 14:50 (as mean folate concentration during this time appears to be representative of mean folate concentrations over a 24-h period) [34, 49], 2–3 h after their last full expression. Participants will completely express breastmilk (manually or by electric pump) from the right breast. Once breastmilk is expressed, women will be instructed to gently swirl the milk to mix it, and then, using a sterile falcon pipette, they will transfer 0.5 mL into two amber cryovials (each containing 0.005 g ascorbic acid for a 1% dilution) and 1 mL into two amber cryovials. All cryovials will be clearly marked at the 0.5- and 1-mL levels. The women will place all vials in a freezing box which will be stored in the freezer for up to 4 days. Samples will be picked up frozen and transferred to the laboratory for storage at − 80 °C until further analyzed.

### Laboratory analyses

Serum folate (nmol/L) and RBC folate (nmol/L) will be analyzed by microbiological assay, as globally recommended, using the microtiter plate method outlined by Molloy et.al [50]. RBC folate (nmol/L) will be calculated with the following formula [51]:

$$ RBC\;\mathrm{Folate}=\frac{\left(\mathrm{Whole}\ \mathrm{blood}\ \mathrm{hemolysate}\ \mathrm{folate}\ast 11\right)-\mathrm{Serum}\kern0.17em \mathrm{folate}\left(1-\mathrm{Hematocrit}/100\right)}{\mathrm{Hematocrit}/100} $$

Plasma biomarkers including UMFA (nmol/L), *S-*adenosyl-methionine (μmol/L), *S-*adenosyl homocysteine (μmol/L), total homocysteine (μmol/L), total cysteine (μmol/L), methionine (μmol/L), free choline (μmol/L) and betaine (μmol/L) will be analyzed using liquid chromatography mass spectrometry [52–54]. Vitamin B_12_ (pmol/L) will be analyzed using an immunoanalyzer. Pyridoxal-5-phosphate (nmol/L) will be analyzed using liquid chromatography-tandem mass spectrometry [55]. Gene variants will be genotyped by using TaqMan SNP genotyping assays [1, 56]. A complete blood count will be performed using an automated hematology analyzer (Sysmex XNL-550). Breastmilk folate forms (including UMFA, THF, 5-methyl-THF, 5-formyl-THF and 5,10-methenyl-THF) will be quantified via liquid chromatography-tandem mass spectrometry; total folate will represent the sum of these forms [33, 52]. Breastmilk FBP concentrations will be measured using a competitive binding radioassay procedure (as described by Selhub et al.) [57].

### Summary of data to be collected

#### Demographic, medical and nutrition data via structured questionnaires

Baseline data collection will include age, ethnicity, parity, education, occupation, household income, medical and medication history, reported pre-pregnancy weight, general diet (including vegan, vegetarian, ketogenic, gluten-free food intake and any foods/food groups avoided for any reason) and supplement use. Endline data collection will include any changes in overall health or medication use since the baseline visit.

#### Anthropometrics

Pre-pregnancy BMI will be calculated using self-reported pre-pregnancy weight and measured height. Participant measurements will be taken at baseline (weight and height) and endline (weight only) for calculation of gestational weight gain throughout the intervention period and total weight gain throughout pregnancy (using self-reported pre-pregnancy weight and measured endline weight).

#### Dietary assessment

Total DFEs (μg/day) from food folates and supplemental folic acid will be calculated using the Block Folic Acid/Dietary Folate Equivalents Screener (NutritionQuest, Berkeley, CA, USA), a validated food frequency questionnaire [45].

#### Postpartum data

Total weeks of supplementing, weeks’ gestation at delivery, compliance to the breastmilk collection protocol, and timing of folate supplementation prior to collection of breastmilk and postpartum blood samples will be recorded.

### Withdrawal criteria

Withdrawal criteria include spontaneous or planned termination of pregnancy, use of additional folate/folic-acid-containing nutritional supplements and following an adverse/serious adverse event associated with the supplements/trial intervention (as determined by the qualified investigator (CM)). Participants may also self-withdraw at any time and no further information will be collected. Regardless of the reason for withdrawal, all previously collected data will be retained for analysis. Participants who give birth prior to their endline visit will have the option to continue in the postpartum phase of the study, given that they continue to supplement daily with the study supplements.

### Data handling and privacy

All data and biological samples will be identified using each participant’s study ID. Data with personal identifiers will be stored on an encrypted, password-protected computer in a secure server space with the BC Children’s Hospital Research Institute. Data with personal identifiers de-linked will be cleaned and double entry will be completed by the research coordinator (KMC) and an independent research assistant into REDCap (Research Electronic Data Capture) hosted by BC Children’s Hospital Research Institute. KMC, JAH and CDK will have access to the final dataset. Paper documents will be stored in a locked filing cabinet and biological samples will be stored in a locked freezer housed inside the principal investigator’s laboratory (CDK) at the University of British Columbia. Participants may be told their folate supplement allocation and blood/breastmilk (if applicable) folate results (including DFEs, serum and RBC folate at baseline, endline and postpartum, and total breastmilk folate concentrations) after final statistical analyses are completed.

### Data and statistical analysis

Descriptive statistics will be used for participant baseline data. Continuous variables will be reported with a mean ± standard deviation (or median and interquartile range if not normally distributed) and categorical variables as absolute numbers per group and percentage. Capsule counts will be used to calculate participant adherence and the supplement diary will used to provide descriptive insight into adherence barriers. Data will be assessed for normality and transformations or use of equivalent non-parametric tests will be considered if data do not follow a normal distribution. Statistical analyses will be performed using Stata (StataCorp, College Station, TX, USA). All analyses will initially be completed on an intention-to-treat basis according to initial group allocation at baseline and without any imputation for missing data. Secondary per-protocol analyses will be conducted including those who have fully completed the study and adhered to the study protocol. Multiple imputation will be used to correct for missing data as appropriate in sensitivity analyses [58].

#### Primary analyses

We will calculate the mean ± standard deviation for primary outcomes (serum folate, RBC folate and plasma UMFA) in each group at baseline and endline, as well as the mean ± standard deviation of the within-woman change in these outcomes in each group. These estimates will be used to inform the sample size calculation of the definitive trial.

#### Secondary analyses

Exploratory analyses of primary outcomes will be conducted using a multiple linear-regression model to investigate whether the change from baseline to endline differed according to treatment status, adjusting for baseline values and explanatory variables (DFEs, exploratory biomarkers, weeks’ gestation at supplement initiation). The proportion of women with UMFA ≥ 0.2 nmol/L (considered a detectable concentration) at baseline and endline in each group will also be calculated. To inform our secondary objective, the overall study participation rate will be estimated by dividing the total number of women enrolled by the total number of women who were interested in participating. Weekly recruitment rate will be estimated by dividing the total number of women enrolled by the total number of weeks that it took to recruit them. Participant retention rate will be estimated by dividing the number of women who complete the full trial by the total number of women enrolled. For each recruitment strategy (including posters, social media, word of mouth), total reach (social media only), inquires, enrollments and cost will be evaluated. The odds of enrollment after inquiring for each strategy will be evaluated with an odds ratio. As available, reasons for not enrolling will be recorded.

#### Postpartum analyses

We will calculate the mean ± standard deviation for proportion of folic acid in breastmilk (dividing concentration of breastmilk folic acid by sum of all breastmilk folate forms) and for maternal postpartum RBC folate. Linear regression will be used to estimate the association between breastmilk folic acid concentration and maternal postpartum plasma UMFA. Postpartum outcomes will be further analyzed using multiple linear regression, adjusting for endline values and explanatory variables (DFEs, total weeks of supplementing, adherence to breastmilk collection protocol).

### Safety

Folic acid is provided at a dose that meets Canadian recommendations for pregnant women (0.4–1.0 mg/day); thus, is considered low risk. The calcium salt of (6*S*)-5-methyltetrahydrofolic acid is recognized as safe and is included in Canadian prenatal multivitamins with no established tolerable upper limit (no risk of harm is currently known). Randomization will occur between 8 and 21 weeks’ gestation (after neural tube closure). Considering the low risk of this pilot study, assembly of a data monitoring committee was not deemed necessary. All safety concerns and, if necessary, the decision to terminate the trial, will be addressed by the principal investigators (CDK and JAH) and a qualified investigator (CM) and reported to the Research Ethics Board and Health Canada. Unblinding of the intervention codes may be undertaken at any time if deemed necessary for participant safety monitoring.

## Discussion

These pilot data are critical to inform the design of a definitive trial which will be powered to assess whether (6*S*)-5-methyltetrahydrofolic acid supplementation is as effective as synthetic folic acid in increasing serum and RBC folate concentrations during pregnancy, while resulting in lower UMFA. Although analyses in the postpartum phase will be exploratory, no other study has assessed the long-term supplementation of folic acid as compared to (6*S*)-5-methyltetrahydrofolic acid starting in mid-pregnancy and subsequent influence on folate concentrations in breastmilk and lactating blood samples; thus, investigation is warranted and may be further explored in the definitive trial. Ultimately, a definitive trial should help to inform the safest and most effective form of folate supplementation for pregnant women and their babies.

## Trial status

*11 March 2020, version 7; as of September 2019, recruitment is ongoing and is expected to be completed April 2021 and version*: 7 March 2020, version 2; as of September 2019, recruitment is ongoing and is expected to be completed August 2020.

## Supplementary information

**Additional file 1.** Standard Protocol Items: Recommendations for Interventional Trials (SPIRIT) 2013 Checklist: recommended items to address in a clinical trial protocol and related documents.

**Additional file 2.** The World Health Organization Trial Registration Data Set

## Data Availability

The datasets used and/or analyzed during the current study are available from the corresponding author on reasonable request.

## Abbreviations

5-MTHF: 5-methyl-tetrahydrofolate
DFEs: Dietary folate equivalents
DHFR: Dihydrofolate reductase
MTHFR: Methylene-tetrahydrofolate reductase
NTD: Neural tube defects
RBC: Red blood cell
UMFA: Unmetabolized folic acid
EDTA: Ethylenediaminetetraacetic acid
BMI: Body mass index
FBP: Folate-binding protein
