# Supplementary material for: Is natural (6S)-5-methyltetrahydrofolic acid as effective as synthetic folic acid in increasing serum and red blood cell folate concentrations during pregnancy? A proof-of-concept pilot study
Source: Trials. 2020 May 5;21:380. doi: 10.1186/s13063-020-04320-3 (PMC7201521; doi:10.1186/s13063-020-04320-3)
Supplement: Supplementary file 2 — Additional file 2. The World Health Organization Trial Registration Data Set [file 13063_2020_4320_MOESM2_ESM.docx]

| **Data Category** | **Information** |
| --- | --- |
| Primary registry and trial identifying number | ClinicalTrials.gov, identifier: NCT04022135 |
| Date of registration in primary registry | July 14, 2019 |
| Secondary identifying numbers | Health Canada, Clinical Trials Application, Notice of Authorization, Submission No. 244456, approved July 26^th^, 2019.  UBC Children’s and Women’s Research Ethics Board (H18-02635). Approved September 16^th^, 2019. |
| Source(s) of monetary or material support | **Monetary:**  Healthy Starts Catalyst Grant provided by the BC Children’s Hospital Research Institute, Vancouver, Canada  Frederick Banting and Charles Best Canada Graduate Scholarship-Master’s from the Canadian Institutes of Health Research  **Material:**  Merck & Cie (Schaffhausen, Switzerland), in-kind donation of (6S)-5-methyltetrahydrofolic acid  Natural Factors (Coquitlam, Canada) in-kind donation of folic acid and prenatal vitamin ingredients and for compounding and packaging all study supplements |
| Primary sponsor | Dr. Crystal Karakochuk |
| Secondary sponsor(s) | N/A |
| Contact of public queries | Crystal Karakochuk, University of British Columbia, Faculty of Land and Food Systems, Food, Nutrition and Health, 2205 East Mall, V6T 1Z4, Vancouver, BC, Canada. Email: [crystal.karakochuk@ubc.ca](mailto:crystal.karakochuk@ubc.ca) |
| Contact for scientific queries | Crystal Karakochuk, University of British Columbia, Faculty of Land and Food Systems, Food, Nutrition and Health, 2205 East Mall, V6T 1Z4, Vancouver, BC, Canada. Email: [crystal.karakochuk@ubc.ca](mailto:crystal.karakochuk@ubc.ca) |
| Public Title | Is natural (6S)-5-methyltetrahydrofolic acid as effective as synthetic folic acid in increasing serum and red blood cell folate concentrations during pregnancy? A proof-of-concept pilot study |
| Scientific Title | Is natural (6S)-5-methyltetrahydrofolic acid as effective as synthetic folic acid in increasing serum and red blood cell folate concentrations during pregnancy? A proof-of-concept pilot study |
| Countries of recruitment | Canada |
| Health condition(s) or problem(s) studied | Pregnancy |
| Intervention(s) | Supplementation with folic acid vs natural folate prenatal vitamins daily for 16-weeks of pregnancy (starting between 8-21 weeks gestation); option for women to continue supplementation until 1-week postpartum (to provide a breastmilk and postpartum blood sample) |
| Key inclusion and exclusion criteria | **Inclusion criteria:** i) pregnant woman (singleton pregnancy); ii) living in the greater Vancouver area; iii) <21 weeks gestation at time of consent; iv) 19-42 years of age; v) able to provide informed consent.  **Exclusion criteria:** i) having a pre-existing medical condition known to impact maternal folate status (malabsorptive and inflammatory bowel diseases, active celiac disease, gastric bypass surgery, atrophic gastritis, epilepsy, advanced liver disease, kidney dialysis, type 1 or 2 diabetes mellitus, sickle cell trait/anemia); ii) lifestyle factors known to impact maternal folate status (current smoking, alcohol consumption, recreational drug use); iii) are medium to high risk for development of an NTD-affected pregnancy (applies to women or their male partner: personal or family history [parents or siblings] of other folate sensitive congenital anomalies, personal NTD history or a previous NTD-affected pregnancy); iv) are taking medications known to interfere with B-vitamin metabolism (Chloramphenicol, Methotrexate, Metformin, Sulfasalazine, Phenobarbital, Phenytoin, Primidone, Triamterene, Barbiturates); v) pre-pregnancy body mass index (BMI) ≥30kg/m^2^; or vi) allergy to any study supplement ingredients. |
| Study type | Pilot study, two-arm, randomized, double-blinded |
| Date of first enrolment | October 6^th^, 2019 |
| Target sample size | 60 |
| Recruitment status | Ongoing, 30 study visits complete |
| Primary outcome(s) | Serum folate, red blood cell folate, unmetabolized folic acid |
| Key secondary outcomes | Recruitment and participation rates  Exploratory biochemical measures: plasma *S-*adenosyl-methionine, *S-*adenosyl-homocysteine, total homocysteine, total cysteine, methionine, vitamin B-12, pyridoxal-5-phosphate, free choline and betaine, breastmilk folate forms, maternal postpartum folate concentrations |
